# Advances in Nanogels for Topical Drug Delivery in Ocular Diseases

**DOI:** 10.3390/gels9040292

**Published:** 2023-04-02

**Authors:** Yongkang Wu, Qing Tao, Jing Xie, Lili Lu, Xiuli Xie, Yang Zhang, Yong Jin

**Affiliations:** School of Pharmacy, Anhui Medical University, No. 81 Meishan Road, Shushan District, Hefei 230032, China; yongkang4216@126.com (Y.W.); taoqing2023@163.com (Q.T.);

**Keywords:** nanogels, ocular diseases, drug delivery systems, natural polymers, contact lenses

## Abstract

Nanotechnology has accelerated the development of the pharmaceutical and medical technology fields, and nanogels for ocular applications have proven to be a promising therapeutic strategy. Traditional ocular preparations are restricted by the anatomical and physiological barriers of the eye, resulting in a short retention time and low drug bioavailability, which is a significant challenge for physicians, patients, and pharmacists. Nanogels, however, have the ability to encapsulate drugs within three-dimensional crosslinked polymeric networks and, through specific structural designs and distinct methods of preparation, achieve the controlled and sustained delivery of loaded drugs, increasing patient compliance and therapeutic efficiency. In addition, nanogels have higher drug-loading capacity and biocompatibility than other nanocarriers. In this review, the main focus is on the applications of nanogels for ocular diseases, whose preparations and stimuli-responsive behaviors are briefly described. The current comprehension of topical drug delivery will be improved by focusing on the advances of nanogels in typical ocular diseases, including glaucoma, cataracts, dry eye syndrome, and bacterial keratitis, as well as related drug-loaded contact lenses and natural active substances.

## 1. Introduction

The eye, an important, intricate, and delicate part of the human body, has a significant impact on the quality of work, study, and life. According to the Lancet Global Health report, in 2020, nearly 433 million people were blind and 553 million had different degrees of vision impairment worldwide [1]. There are substantial challenges for people when it comes to preventing and treating ocular diseases.

Topical drug delivery is the most popular noninvasive method for the treatment of ocular diseases [2], though it must be able to reach the treatment area, provide adequate doses of drug ingredients, and maintain them in the eye continuously. Eye drops are traditionally the first option for therapy for many anterior segment ocular diseases including glaucoma, cataracts, dry eye syndrome, and inflammatory infections. Their products make up about 90% of the worldwide ocular drug market [3]. Even so, it is quite interesting to observe that even after topical application to wet the eye and then after different elimination effects, eye drops, which are used by such a wide variety of individuals worldwide, only have a bioavailability of 5% [4]. This combination of anatomical (cornea, conjunctiva, blood-aqueous, and blood-retinal barriers) and physiological (eyelid movement, tear flow, nasolacrimal drainage), together with protein binding, systemic absorption, and enzymatic degradation, causes such low pharmacokinetic parameters [5,6,7]. In spite of being applied topically in tiny dosages, eye drops are largely absorbed into the veins of the nasolacrimal and nasopharynx mucosa which bypass hepatic first-pass metabolism, perhaps increasing drug blood plasma concentrations to the point that systemic side effects are possible [8]. Traditional ocular formulations are commonly available on the market in addition to eye drops and include ointments, suspensions, lotions, and so on. Generally speaking, they prolong drug retention time in the eye and enhance the bioavailability, yet they have drawbacks as well. Due to the bases of the ointment being a semisolid preparation, and that tears have different refractive indices, they often cause blurred vision and may result in inaccurate dosage [9]. To increase its effectiveness, it is typically advised that patients apply it before sleeping. The problem with suspensions is that they are unstable by nature, and the crystal structure of that structure could change if placed somewhere for a while. This could alter particle size, thus affecting their solubility by either increasing or decreasing it [10]. Moreover, increased tear secretion and rapid drug drainage from the individual’s ocular surface due to ocular irritation by the presence of particles > 10 μm in solution greatly reduced the bioavailability [11]. Eventually, traditional ophthalmic formulations will have lower pharmacological effects after drug delivery, requiring increased dose frequency, and patients may struggle to adhere to treatment regimens carefully. In addition, various additives, including permeation enhancers, viscosity enhancers, and cyclodextrins, are usually added to ophthalmic formulations to increase their therapeutic efficacy [12]. This makes people more cautious in their choices and may also add additional side effects such as ocular irritation, visual disturbances, inflammation, and instabilities. The prevalence of traditional ophthalmic preparations, whose most fundamental advantages are their ease of production, attractive price, and convenience of application, is unshakable, despite unsatisfactory outcomes. In particular, people find these preparations easier to use, and are more patient compliant, compared to intraocular injection operations since they have fewer side effects than systemic administration or vitreous or periocular injections that require some skilled medical workers.

In recent years, scientists have investigated and created many novel types of ophthalmic formulations, including micelles, liposomes, nanoparticles, nanosuspensions, dendritic polymers, microneedles, cubosomes, niosomes, nanowafers, etc., providing a number of alternatives to the drug delivery of traditional ophthalmic formulations [13]. Nanogels are formed from natural polymers, synthetic polymers, or a combination thereof, and their three-dimensional network structure allows for the encapsulation of small molecules, oligonucleotides, and even proteins [14], whose unique characteristics make it possible for drug delivery, diagnostics, and imaging and have great potential for application in the biomedical, tissue engineering, and pharmaceutical fields [15,16,17,18,19]. Recently, the application of nanogels in the eye has attracted more attention, not only as an upgrade to hydrogels or microgels [20] but also as a nanocarrier that can be combined with micelles, liposomes, dendritic polymers, etc., which are superior alternatives since they have higher biocompatibility, drug loading capacity, and lower toxicity [21,22,23]. Moreover, studies have found that simply increasing the viscosity of the drug solution does not necessarily increase its retention time on the ocular surface. Nanogels made of nanoparticles and gel materials have higher adhesion and mechanical strength than pure gel [24]. Despite that, there have been a few reports regarding the use of nanocarriers or normal gels in the eyes. Those have mainly focused on the characteristics of various nanocarriers, with nanogels receiving little attention. This review aims to focus on ocular diseases related to the application of nanogels for topical drug delivery. In this way, more scholars will become interested in nanogels as novel carriers for topical drug delivery, expanding their horizons on a larger scale.

## 2. Ocular Structure and Barriers

**Figure 1:**
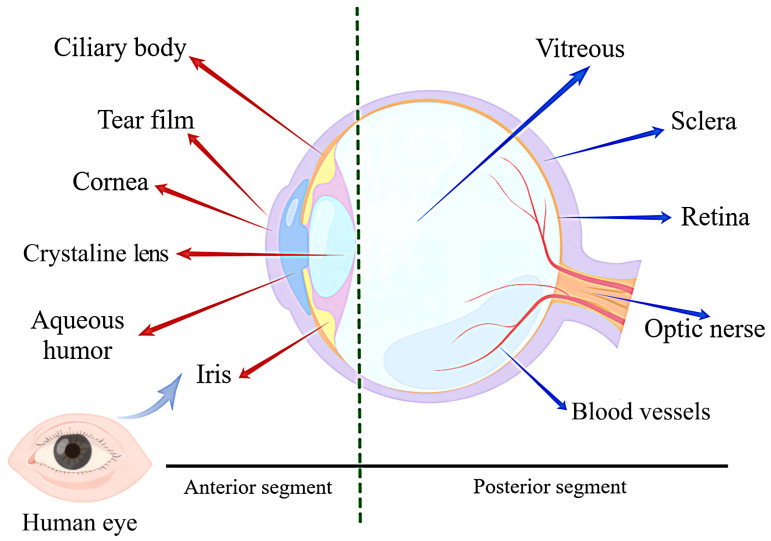
Schematic representation of the ocular structure. The eye consists of two parts: the anterior segment and the posterior segment. The anterior segment includes the cornea, conjunctiva, iris, ciliary body, crystalline lens, and aqueous humor; the posterior segment includes the sclera, choroid, retina, and vitreous humor. They are the main barriers to ocular drug delivery.

The human eye has a delicate anatomy that is broadly divided into two parts, the anterior and posterior segments. The former includes the cornea, conjunctiva, iris, ciliary body, crystalline lens, and aqueous humor, while the latter includes the sclera, choroid, retina, and vitreous (Figure 1) [2]. The anterior and posterior segments are affected by different pathogenic factors [13,25], resulting in different types of diseases, making ocular drug delivery one of the most challenging tasks for researchers [26]. The following is a brief overview of the relevant ocular structures that affect drug delivery.

The cornea, which is located at the front of the eye, is transparent and round in shape and is primarily composed of avascular connective tissue [27]. In addition to protecting the eye from foreign microorganisms, the cornea is known as the largest static barrier that restricts drug molecules from entering the eye [28]. Physiologically, the cornea is divided by scholars into five layers: the epithelium, Bowman’s membrane, stroma, Descemet’s membrane, and inner endothelium [29]. The epithelium and endothelium are made up of multiple layers of cells that prevent hydrophilic molecules from entering the aqueous humor while allowing lipophilic molecules to pass through, whereas the stroma between the epithelium and endothelium is made up of hydrophilic collagen and thus prevents the passage of lipophilic molecules while allowing the osmotic diffusion of hydrophilic molecules [30]. The “sandwich” structure of the cornea, which is hydrophobic on both sides and hydrophilic in the middle, requires a large distribution coefficient of drugs in both the water and oil phases, so it also determines the physical and chemical properties of drugs entering the cornea. In addition, the cornea has a mechanism for the self regulation of pH, known as the “tear buffer system”. The acid–base balance on the corneal surface will be broken, and ocular diseases may further deteriorate when the quantity or quality of tear fluids is decreased.

In the outermost layer of the cornea, the tear film, which is the most important dynamic barrier for ocular drug delivery and is composed of lipid, aqueous, and mucin layers, the main function of which is to nourish and protect the cornea [31,32]. The aqueous layer, which is 7–8 μm thick, makes up the majority of the tear film. The active drug can be bound to and metabolized by proteins and enzymes in the aqueous phase, which decreases its bioavailability for the eyes [33].

The conjunctiva is a fragile, thin, translucent, and vascularized mucous membrane in the front third of the eyeball that is more permeable than the cornea and has 22 times the surface area, allowing for more absorption of the drug [9,34,35]. However, due to the presence of conjunctival capillaries and lymphatics, drug absorption in the conjunctiva is generally considered futile [29]. Large quantities of drugs are lost in the systemic circulation, which reduces the bioavailability of ocular drugs and may cause systemic adverse reactions. The human conjunctival sac can hold approximately 30 μL of tear fluid transitorily, but the typical tear volume is only 7 μL. Tear fluids have a high turnover rate, limiting the retention time of ocular drugs [36].

The retina is a soft, transparent sense organ located in the innermost layer of the human eye, which consists of multiple layers of membranes with a mean thickness of 249 μm, with complex functions of photoconversion and transmission [37,38]. Not only that, but the retina, as part of the vital architecture of the blood-retinal barrier, can protect itself from potentially harmful substances in the blood. On the other hand, it becomes a significant barrier to ocular drug delivery, with larger drug molecules attempting to reach the retina with difficulty [29].

The crystalline is a transparent tissue made up of lens epithelial cells (LECs) and lens fiber cells that are encased by the lens capsule [39]. The ability of humans to see clear images depends on the lens in the anterior segment of the eye and the cornea to focus light from objects on the retina in the posterior segment of the eye. Loss of lens transparency results in damage to vision.

## 3. Nanogels System

### 3.1. Concept and Properties of Nanogels

In recent years, nanotechnology has rapidly developed and been widely used in drug delivery systems, where nanocarriers have gained popularity in biomedicine [40]. The development of nanocarriers such as nanoemulsions, liposomes, nanomicelles, nanosuspensions, nanoparticles, and nanoemulsions offers more options for the prevention, diagnosis, and treatment of ocular diseases [41]. Nanoscale size has many advantages in drug delivery processes, such as an improved solubility of hydrophobic drugs [42]; the enhanced permeability and retention (EPR) effect-mediated passive targeting of drug delivery to increase drug accumulation in tumor tissue [43]; functionalization of their structures with various receptor ligands for specific applications [44]; and site-specific and rate-controlled drug delivery strategies mediated by stimuli-responsive nanoparticulate, et al. [45]. Nanogels, the concept of which was first proposed by Vinogradov et al. [46] in 1999, are nanoscale particles prepared by crosslinked polymer three-dimensional networks [47]. The particle size of nanogels can reach several hundred nanometers compared to microgels (above 1 μm) and macrogels (above 100 μm), and the EPR effect, which makes them ideal for delivering nucleic acid-based drugs to cells [48]. Nanoparticles typically include lipid-based, inorganic-based, carbon-based, polymeric, and biomimetic nanoparticles from a material standpoint. Under this definition, it is obvious that nanogels are polymer nanoparticles [49]. They have a strong ability to absorb and retain large amounts of liquid due to the long polymer chains of nanogels that are distributed with various hydrophilic groups such as -OH, -COOH, -CONH_2_, and -SO_3_ H [50]. When the fluid penetrates, it instantly swells but does not dissolve [15].

The combination of gels and nanoparticles shows a promising strategy that is regarded as a major breakthrough in the field of drug delivery [51]. With controllable drug release characteristics, nanogels differ from typical nanoparticles that have an adjustable particle size, shape, and sensitivity to other microenvironment stimuli, including pH, temperature, ionic strength, and proteases. Also, unlike ordinary nanocarriers, nanogels have relatively high drug encapsulation efficiency, biocompatibility, and simplicity of preparation [16]. In addition, They can incorporate the drug on their own or in combination with liposomes, solid lipid nanoparticles, dendrimers, or polymeric nanoparticles [52]. Based on the excellent properties listed above, nanogels are an ideal nanocarrier for drug delivery and have been regarded as the next generation of drug delivery systems with the potential to completely change the way therapy drugs affect patients’ lifestyles [53]. Such advantages of nanogels have been applied to drug delivery systems, which have produced important developments in efforts to find solutions to the problem of ocular topical drug delivery. Many studies have found promising applications for nanogels in the treatment of widespread ocular diseases such as glaucoma, dry eye syndrome, cataracts, bacterial keratitis, inflammatory infection, and corneal wound healing.

### 3.2. Preparations of Nanogels

A variety of methods can be used for the preparation of nanogels. Given the scope of this review, it is not appropriate to discuss the preparation of nanogels in depth, but only briefly summarize.

**Figure 2:**
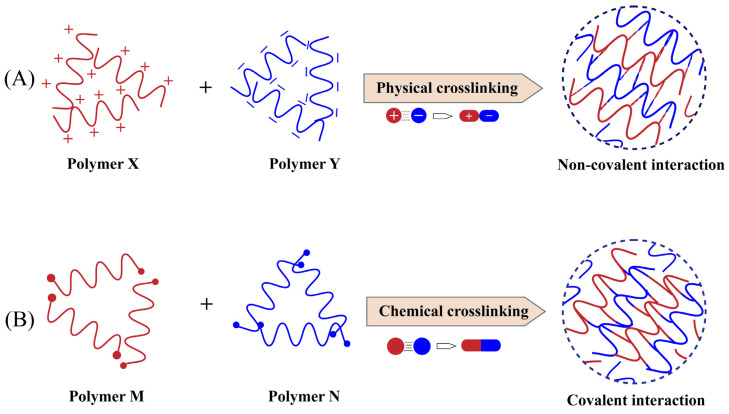
The preparation of nanogels is divided into two categories based on the type of bonds in the polymer network. (**A**) polymer X and polymer Y form physical crosslinked nanogels through noncovalent interactions, which are characterized by simple but unstable preparation; (**B**) polymer M and polymer N form chemically crosslinked nanogels through covalent interactions, which are characterized by stable structures but require safety considerations.

The essence of nanogel preparation is the formation of suitable crosslinks between polymers, and there are two main categories based on the type of bonds present in the polymer crosslinking network: chemically crosslinked nanogels, where crosslinks are formed by covalent bonds, and physically crosslinked nanogels, where self-assembly is formed by weak bonds with noncovalent bonds (Figure 2) [54]. Physical crosslinking relies on noncovalent interactions, mainly hydrogen bonding, van der Waals forces, hydrophobic interactions, electrostatic interactions, etc. [55], which do not require complex reactions, and the synthesis process is flexible and convenient. In addition, nanogels formed by hydrophobic interactions can encapsulate various types of hydrophobic drugs or biological macromolecules such as protein and peptide molecules [56]. Due to their lower binding energy, physically-crosslinked nanogels are more fragile and have an unstable tendency in blood circulation, however, which is generally negligible when discussing topical use in the eye. Chemical crosslinking is a more common strategy in nanogel preparation and is classified as emulsion polymerization, reversible addition-fragmentation chain transfer (RAFT), click chemistry crosslinking, and photo-induced crosslinking [16]. Chemically-crosslinked nanogels have higher stability in general, though the disadvantage is that chemical crosslinking agents are usually added, and the toxicity of these crosslinking agent residues after preparation may affect drug delivery safety.

Crosslinking is only one part of nanogel preparation, and polymerization is also necessary. Li et al. [57] In their report, they divided the nanogel preparation process into two main categories: (I) polymerization and crosslinking occur simultaneously; (II) polymerization occurs first, followed by crosslinking. The former method is effective in controlling the size and shape of nanogels, while also enhancing their uniformity and stability. Meanwhile, the latter approach is particularly suitable for preparing nanogels based on natural polymers. Natural polymers with biocompatibility and biodegradability, such as methylcellulose, hyaluronic acid, chitosan, and sodium alginate, have a wide range of applications in the biomedical field, including tissue engineering, antimicrobial applications, and drug delivery [58,59,60,61,62,63]. Some nanogels are synthesized by graft polymerization and hydrophobic modification of natural polymer molecular chains, followed by further crosslinking in aqueous solutions. For example, the potential toxicity and nonbiodegradability of the synthetic polymer N-isopropylacrylamide (PNIPAAM) restrict its application as a nanogel. However, nanogels are formed by combining PNIPAAM with natural polymers, which improve not only biocompatibility and biodegradability but also drug delivery efficiency [64,65].

### 3.3. Stimuli-Responsive Nanogels

**Figure 3:**
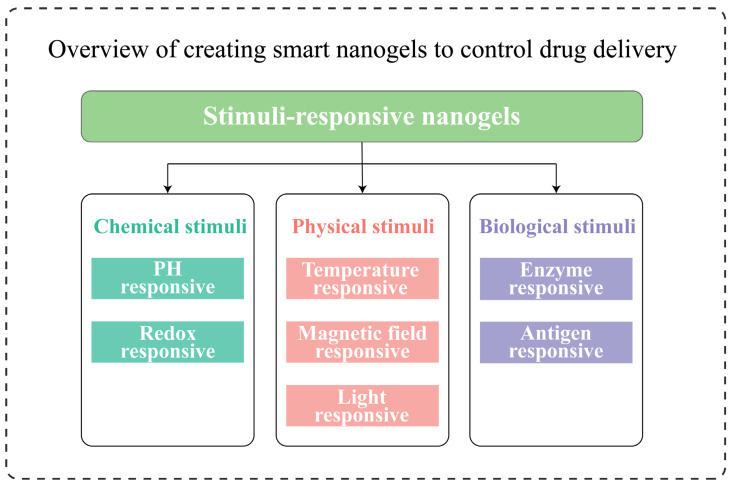
Overview of creating smart nanogels to control drug delivery. According to their response mechanisms, smart nanogels are classified as chemical, physical, or biological stimuli. Synthetic nanogels are chosen to control drug release based on material properties, disease environment, and drug application. However, for the eye, only temperature, pH, and enzyme responses are currently available.

Stimuli-responsive behavior is the ability to show sensitivity to endogenous biochemical stimuli and exogenous physical stimuli. Nanogels based on stimuli-responsive models, also known as “Smart” nanogels [66,67,68], have emerged as a promising prospect in drug delivery systems. As shown in the figure (Figure 3), various types of smart nanogels are outlined, and depending on the disease category and environmental change, these are possible methods currently used for controlled drug release [19]. Smart nanogels are generally prepared with the same technology as normal nanogels, except that the former is created by polymerizing desired functional monomers or functionalized natural polymers and/or combining them with other synthesis strategies followed by cross linking [69].

**Figure 4:**
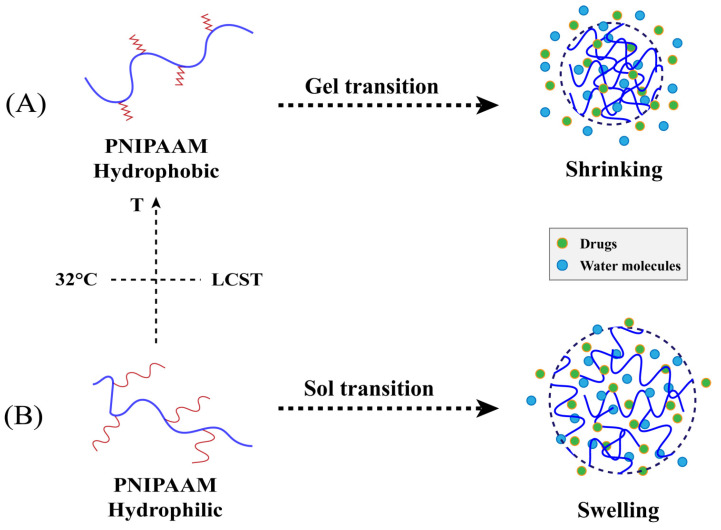
Schematic representation of drug release from temperature-responsive nanogels based on PNIPAAM. (**A**) PNIPAAM above 32 °C, which is above LCST, and nanogels are gel phases, show hydrophobic properties, shrink and release drug; (**B**) PNIPAAM below 32 °C, which is below LCST, nanogels are sol phases, show hydrophilic properties and swell, and release the drug slowly.

Temperature changes are common stimuli, and temperature-responsive nanogels can respond to temperatures higher than the lower critical solution temperature (LCST). In theory, when the temperature is below LCST, the polymer is in a swelling state as a result of the formation of hydrogen bonds between water and the hydrophilic part of the polymer. When the temperature is above the LCST instead, hydrophobic interactions between the hydrophobic parts of the polymer dominate, leading to hydrogen bond breakage and volume phase-transition shrinkage [70]. This kind of polymer is positive-temperature sensitive. However, there are some polymers that show the complete opposite of the former, which is negative temperature sensitivity [66]. The LCST of polymers can be altered by changing the proportion between their hydrophilic and hydrophobic parts [71]. Thermosensitive polymers such as chitosan, poloxam, xyloglucan, methylcellulose, and PNIPAAM are frequently used in ocular drug delivery [72]. Of these, the most widely used polymer is PNIPAAM, which has an LCST of 32 °C in an aqueous solution. PNIPAAM-based nanogels undergo a hydrophilic to hydrophobic and sol-gel transition upon temperature change when used for the eyes. The structural shrinkage of which leads to the release of drug molecules, which could prolong drug retention time in the cornea, and increase ocular bioavailability (Figure 4).

Under normal conditions, the pH of the healthy human eye is 7.4, which is very close to that of the blood. Inducing drug delivery by responding to pH changes is an ideal method to enhance topical drug delivery [73]. pH-sensitive polymers are polyelectrolytes with acidic or basic groups that accept or release protons in response to pH changes in their surroundings [74]. Polyacrylic acid (PAAc) is the most frequently used pH-sensitive polymer in ocular formulations [75], whose aqueous solutions are less acidic and viscous, as well as undergoing sol-gel transition upon pH change.

The nanogels discussed above are merely polymeric nanodelivery systems with a single response mechanism. However, researchers’ primary focus has gradually shifted toward the development of dual-stimulation and multistimulation-responsive nanocarriers that combine multiresponsive capabilities in a single system [69]. For example, Kim and coworkers [76] designed and developed the dual-temperature and pH-responsive nanogel based on PNIPAAM and acrylic acid (AAc), which have proven to have efficient sensitivity to dual stimuli for controlled drug release. For ocular diseases, temperature- and ph-responsive nanogels are more widely studied, in addition to nanogels that are sensitive to redox, enzymes, and photos [19]. However, research articles on the applications of these nanogels in the eye are relatively few and need to be investigated further by pharmacologists.

## 4. Advances in Nanogels for Ocular Diseases

Topical delivery of nanogels is commonly used to treat common anterior segment diseases such as glaucoma, cataract, dry eye syndrome, and bacterial keratitis. Furthermore, nanogels can be used to prepare contact lenses and load some natural bioactive substances with high toxicity and poor stability but anti-inflammatory and antioxidative properties for ocular therapies. Table 1 displays the advances in nanogels for ocular diseases and lists their applications, polymer composition, drug loading, and preparation methods.

### 4.1. Glaucoma

Glaucoma is a disease characterized by elevated intraocular pressure (IOP). Increased IOP and IOP variability are now recognized as important risk factors for the development and progression of glaucoma [96]. Glaucoma sufferers are predicted to number 111.8 million by 2040 [97]. Glaucoma treatment usually begins with topical drugs. These current treatments aim to lower or control IOP. Popular drugs include β-blockers, α-agonists, carbonic anhydrase inhibitors, prostaglandin analogs, and cholinergic drugs [98], though their bioavailability is limited. The main obstacles to treating glaucoma are patient compliance and drug efficacy, which are being further questioned by frequent daily dosing [99,100,101].

Timolol maleate (TM) is a glaucoma treatment drug, but it is not the first choice since it lacks an ideal therapeutic index and must be administered multiple times daily. In addition, TM as a β-blocker could have serious systemic side effects. In this case, the application of nanocarriers, which can be loaded with ocular drugs and continuously released over a long time, has become an alternative solution to the problem. Encapsulation of TM in nanocarriers such as nanoparticles [102], liposomes [103], cubosomes [104], and nanogels has become a strategy for prolonging ocular drug retention time. Ilka et al. [77] prepared the TM-loaded nanogels based on natural chitosan polymers, whose better-targeted delivery and controlled release could improve drug pharmacokinetics and cytotoxicity, thereby increasing TM effectiveness and corneal permeability. The drug release curve indicated that the permeability of the nanogel was significantly increased, approximately twice that of ordinary TM eye drops. Furthermore, the system is stable and suitable for TM glaucoma treatment. Cuggino’s group [78] created novel nanogels based on PNIPAAM and AAc by precipitation and dispersion free-radical polymerization, which are efficiently loaded with TM through ionic interaction, have excellent dispersion in simulated tear fluid and are the ideal size for topical applications. The results of the in vitro release curve showed that the nanogels can deliver TM in a sustained manner and keep IOP in the normal range for two days with only one application.

Pilocarpine is commonly used to treat open angles and other types of chronic glaucoma. The main problem with pilocarpine drops is that the drug is released immediately after administration, followed by a rapid decline to insignificant levels within minutes. To improve the ocular bioavailability of pilocarpine, Abd El-Rehim et al. [79] prepared the PVP/PAAc nanogels based on pH-sensitive polyvinylpyrrolidone (PVP) and PAAc by γ radiation-induced polymerization, and pilocarpine was loaded into the nanogels through electrostatic interactions. In vitro release studies showed that pilocarpine-loaded PVP/PAAc nanogels released slowly compared to the sample solution, with only about 10% of the drug released within the first 15 min and about 55% released within 6 h. Furthermore, the nonirritating, adhesive, and rheological properties of PVP/PAAc nanogels, as well as their slow release properties, prove the potential for improving the therapeutic qualities and bioavailability of pilocarpine.

Acetazolamide (ACZ) is a well-known carbonic anhydrase inhibitor that significantly reduces IOP. However, due to the high oral doses of acetazolamide required for therapeutic effects, it often causes harmful systemic side effects such as nephrotoxicity and metabolic acidosis [105]. Abdel-Rashid and colleagues [80] obtained chitosan–sodium tripolyphosphate and ACZ-loaded CS-TPP nanogels by an ionotropic gelation process, which has good mucosal adhesion and provides effective local ACZ delivery. In vivo experiments showed that CS-TPP nanogels were the least irritating compared to topical ACZ suspension and also significantly prolonged the effect of IOP reduction compared to oral ACZ tablets. These results demonstrate that ACZ-loaded CS-TPP nanogels are safe and efficient for the local treatment of glaucoma.

### 4.2. Cataract

Cataracts are one of the leading causes of blindness in the world, and surgery is the most popular method [106]. However, a large number of patients have visual impairment as a result of postoperative residual LEC migration and proliferation, as well as their differentiation into fibroblasts and lens fiber-like cells [107]. This postoperative complication, known as posterior capsular opacification (PCO), affects 20–40% of adult cataract patients and has no clinically-proven pharmacological treatment [108]. Intraocular lens (IOL) implanted in the lens capsule after cataract surgery as an alternative organ to prevent PCO is a simple and effective method that has shown promise in clinical applications [109]. Since it is implanted directly into the eye, the IOL as a drug delivery device for PCO treatment can increase drug bioavailability and patient compliance [110].

Nibourg and colleagues [81] prepared nanofiber-based nanogels that were used as an extracellular environment for LECs to modulate PCO response after lens surgery in a porcine eye model, from which the lenses were extracted for culture for three weeks. Compared with the hyaluronic acid-filled lens control sample, the nanogels maintained normal epithelioid morphology of LECs and showed less alpha smooth muscle actin expression, inhibiting PCO formation. In addition, the results show that the presence of nanogels improved cell adhesion. Gautam et al. [82] designed acrylic matrix nanogels as intraocular lens analogs and proportionately implanted them in the capular bag of the mouse lens after performing a mock cataract operation. A hydrophilic crosslinked acrylate nanogel was used to coat substrates to elute sorbinil, an aldose reductase inhibitor, which has been shown in the past to inhibit PCO. Analysis of postoperative mouse eyes showed that the drug delivery device was able to deliver an amount of PCO inhibitor sufficient to inhibit PCO markers within five days. Nanogels may provide an effective means of delaying or preventing PCO development by facilitating the delivery of PCO inhibitors to the postoperative lens capsule.

### 4.3. Dry Eye Syndrome

Dry eye syndrome (DES), also known as dry keratoconjunctivitis, is a complex ocular disease caused by multiple factors. DES is characterized by loss of tear film homeostasis and a lack of or excessive evaporation of tears, resulting in an inability to provide adequate lubrication to the eye. This causes eye discomfort, tissue damage, and even severe vision loss, seriously affecting people’s normal lives [111]. Traditional DES eye drops require high doses and frequent dosing due to their insufficient precorneal retention time, which makes treatment results less than ideal. DES is associated with tear fluid, which is essential to nourish and protect the ocular surface, so the topical application of tear substitutes has become the first line of treatment for DES [112].

PAAc is widely used as a tear substitute due to its excellent mucosal adhesion and high-water absorption. When applied topically to the eye, it immediately forms a lubricating film on the conjunctiva and cornea that lasts for a long time. The disadvantage is that the viscosity is high under physiological conditions, resulting in blurred vision and difficulty blinking [113]. Nanogels based on PAAc for a tear substitute are a practical way to overcome the shortcomings. PVP/PAAc nanogels, previously used in glaucoma [79], take on a new role in Swilem et al. [83]. PVP/PAAc nanogels were prepared using ionizing radiation and topical application to a DES model. When compared to a commercial tear substitute, a highly viscous Vidisic^®^ gel, the experimental results showed that twice-daily drops of the PAAc-rich nanogels formulated with 20 kGy were more effective, improved DES more quickly, and enhanced efficacy and compliance.

**Figure 5:**
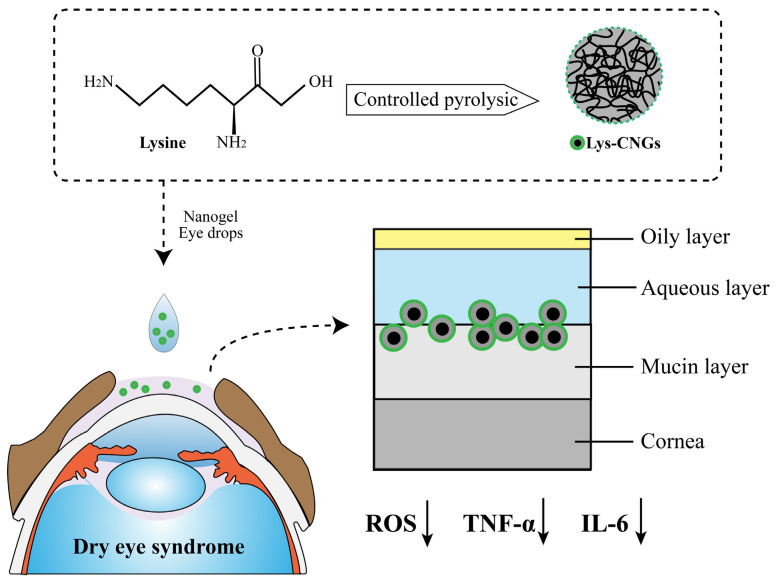
Lysine-carbonized nanogels (Lys-CNGs) were prepared as eye drops to treat dry eye syndrome (DES) through controlled pyrolysis of lysine. Lys-CNGs inhibit the overexpression of ROS and proinflammatory cytokines TNF-α & IL-6, which have significant effects on the development of DES. In addition, Lys-CNGs penetrate into the mucin layer to prolong retention time at the ocular surface.

Reactive oxygen species (ROS) are byproducts of mitochondrial respiration [114,115,116]. A large number of studies have shown that oxidative stress caused by ROS imbalance is critical in the pathogenesis of DES. Many carbon-based nanomaterials can play a role as electron donors and acceptors, as well as pro-oxidants and antioxidants [117]. Lin and colleagues [84] used controlled pyrolysis of lysine hydrochloride to compose carbonized nanogels (Lys-CNG) with strong antioxidant and anti-inflammatory properties for the long-term treatment of DES (Figure 5). High biocompatibility of Lys-CNG was shown in both in vivo and in vitro experiments. Topical eye drops formulated from Lys-CNGs effectively alleviated DES in rabbit eye models; their cationic and crosslinked polymeric features also prolong precorneal retention time and improve ocular bioavailability. Lys-CNG has strong mucosal adhesion and good biocompatibility, and it can achieve the ideal therapeutic effect in the treatment of DES without increasing the drug dose, so it has the potential to be used in the future as long-lasting DES drops.

### 4.4. Bacterial Keratitis

Keratitis is caused by a variety of factors, including inflammatory infections and microorganisms. There is currently no clinical consensus on the criteria for defining keratitis, and there are a variety of criteria [118]. This review discusses bacterial keratitis by using pathogen origin as a diagnostic criterion. Pseudomonas aeruginosa, Staphylococcus aureus, and Streptococcus pneumoniae are common bacterial keratitis pathogens that produce proteins that cause direct or indirect corneal damage [119].

In situ gels are similar to nanogels in that they are composed of environmentally sensitive polymers [120]. Moreover, when in situ gels are dropped into the eye, they are liquid and then rapidly gel in the cul-de-sac of the eye, forming a viscoelastic gel that slowly releases the drug [121]. Davaran et al. [85] combined the thermosensitive polymer PNIPAAM with the pH-sensitive polymer methacrylic acid to develop a dual-stimulated, thermo/pH-responsive in situ nanogel for the delivery of ciprofloxacin (CIP). This in situ nanogel can show the behavior of the in situ gel, an initially transparent solution that transforms into a gel at pH 7 and above 36 °C to prolong the topical delivery of CIP. In addition, the impact of this novel gelling system on bacterial growth and drug resistance development was investigated. CIP release from nanogels was evaluated at the lowest inhibitory concentration and demonstrated an improved antibacterial activity with a potential for topical application in the treatment of bacterial keratitis.

In recent years, bacterial keratitis caused by methicillin-resistant *Staphylococcus aureus* has shown resistance to first-line antimicrobial drugs (e.g., fluoroquinolones), raising concerns [122,123]. Considering the broad-spectrum antimicrobial activity of antimicrobial peptides and the uniqueness of membrane interactions, there is a strong interest in antimicrobial peptides as novel antimicrobial drug [124]. Nanogels are promising carriers for antimicrobial peptides but have not been widely used for antimicrobial applications. The L12 peptide is a highly effective synthetic antimicrobial peptide against gram-positive and gram-negative pathogenic bacteria [125]. During nanogel self assembly, Obuobi et al. [86] use electrostatic interactions between DNA monomers and charged AMP residues to spontaneously encapsulate the L12 peptide. Nanogels (L12-NG) have the ability to continuously deliver L12 peptides. The nanogels for topical delivery to the eye were subtly modified from the DNA hydrogel previously engaged in research for skin-wound treatment by their group [126]. L12-NG sustained release has been shown to have significant anti-S. aureus efficacy both in vitro and in vivo. It was observed that 0.3% L12-NG was clinically superior to an equivalent dose of the fluoroquinolone antibacterial drug gatifloxacin in reducing the number of bacteria after 48 h. Furthermore, the nanogel-loaded antimicrobial peptide strategy is a promising candidate for the clinical treatment of bacterial keratitis due to low drug toxicity and excellent ocular tolerability.

When treating severe bacterial keratitis clinically, it is critical to not only treat the bacterial infection but also to prevent inflammatory damage in the cornea caused by oxidative stress. Lin et al. [87] created carbonized nanogels (Qu/Lys-CNG) with antibacterial and antioxidant properties by heating a mixture of quercetin and lysine in a single step. Quercetin is a natural bioflavonoid that is commonly used as an antioxidant and also has some antibacterial properties [127]. In comparison to free quercetin, Qu/Lys-CNG has superior antibroad-spectrum bacteria (including multidrug-resistant bacteria) and excellent antioxidant capabilities. Qu/Lys-CNG has a dual function that has been shown to be more effective than commercially available antibacterial eye drops in treating bacterial keratitis. Therefore, Qu/Lys-CNGs have great potential for clinical application in bacterial keratitis.

### 4.5. Nanogel Contact Lenses

Contact lenses can be used not only for refractive or cosmetic purposes but also as a drug delivery device for extended-ocular drug delivery. Due to their proximity to the cornea, contact lenses offer some unique advantages in drug delivery and may be a more effective method of drug delivery than traditional eye drops, which are less effective. The presence of a thick layer of fluid between the lens and the cornea, known as the postlens tear film (POTF) [128], results in a retention time of more than 30 min for drugs released from the lens and has a 50% increase in bioavailability compared to about 5 min for eye drops [129,130]. More importantly, therapeutic contact lenses are especially convenient for patients with chronic ocular diseases, such as glaucoma, corneal ulcers, or infections, who have difficulty adhering to the repeated dose regimens of traditional eye drops. Researchers have repeatedly attempted to immerse hydrophilic lenses in drug solutions for drug delivery, but the problem is that the loaded drug is released quickly, and more research is needed to determine how to prolong the drug release time. Contact lenses have been loaded into various carrier systems such as cyclodextrin [131], microemulsion [132], micelles [133], liposomes [134], vitamin E [135], or using molecular imprinting techniques to achieve sustained drug delivery [136]. These approaches enable drug delivery in contact lenses, but commercialization is still limited because critical properties such as contact lens transparency, mechanical properties, water content, and ionic permeability also affect drug release, patient acceptance, and the wearing experience [137,138].

**Figure 6:**
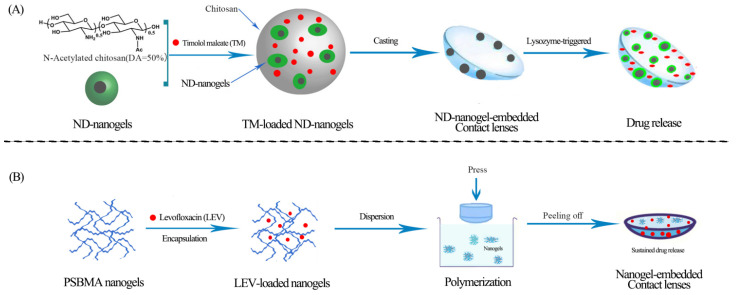
Flowchart for the preparation of two types of drug-loaded nanogel contact lenses (**A**) Nanodiamond (ND) nanogels are crosslinked with chitosan and loaded with timolol maleate (TM), then embedded in a hydrogel and cast into enzyme-responsive contact lenses. The nanogel contact lenses release TM through lysozyme-triggered mechanisms; (**B**) levofloxacin (LEV) is encapsulated in poly (sulfobetaine methacrylate) (PSBMA) nanogels to form LEV-loaded nanogels, which are then dispersed in premonomer solutions, polymerized, and pressed into a mold to obtain nanogel contact lenses that sustain drug release.

Stimuli-responsive contact lens drug delivery is a novel technology that allows for therapeutic dosages on demand while also preventing drug loss due to lens elution during transport and storage [139]. Nanogels not only respond to stimuli, but also have high water absorption, high drug-loading efficiency, and high colloidal system stability, making them suitable for drug-loaded contact lenses. Kim et al. [88] developed a nanodiamond (ND)- nanogel-embedded contact lens with lysozyme-triggered TM release for ocular therapy (Figure 6A). The experimental data show that these contact lenses have the appropriate transparency, water content, mechanical properties, and other key characteristics, and are particularly promising for application and development. Lee and colleagues [89] created thermosensitive, TM-loaded, bicontinuous-microemulsion contact lenses. Later, based on nanogel contact-lens research, the group proposed a new optical IOP measurement method in which IOP changes in a rabbit-acute glaucoma model could be measured by imprinting moiré patterns on a single contact lens when combined with a computer-generated virtual image [140]. These studies are meaningful and suggest that, in the future, the development of “smart” nanogel contact lenses that deliver on-demand drug delivery through perceived IOP may be feasible. Wang et al. [90] reported the preparation of nanogel contact lenses for extended drug delivery time. They synthesized zwitterionic and levofloxacin-loaded nanogels based on poly(sulfobetaine methacrylate) which were fabricated by the one-step reflux-precipitation polymerization method (Figure 6B). In addition, the drug-loaded zwitterionic nanogel contact lenses have excellent antifouling and biocompatible properties [141,142]. Experimenters investigate its structure, water content, light transmission, and mechanical properties and find that these key lens properties are within the recommended range of values for commercial contact lenses. Furthermore, the cytocompatibility of the nanogel contact lenses suggests that they have great potential for long-term oculopathy treatment as an alternative to traditional ophthalmic preparations.

### 4.6. Other Ocular Applications

Instead of focusing on specific ocular disorders, some scholars have investigated the potential common function of a number of eye-beneficial drugs or natural active substances in nanogels.

Jamard et al. [91] prepared dexamethasone-loaded nanogels based on grafting poly(N-tert-butylacrylamide) side chains on methylcellulose through cerium ammonium nitrate to reduce the dose and frequency of administration of traditional eye drops, which improves local ocular therapy. Since nanogels are hydrophobically modified methylcellulose on the outside, the colloidal structure is more stable. The drug releases slowly over a few weeks, according to the results of the experiments, and the rate of release can be controlled by changing the degree of hydrophobic modification.

There is ample evidence that hyaluronan increases drug retention time on the corneal surface and is widely used as a lubricant in ocular surgery and DES treatment, as well as in eye drop formulations [143,144]. Zoratto et al. [92] prepared hyaluronan-cholesterol (HC-NG) nanogels by chemical self-assembly, which effectively loaded hydrophobic and hydrophilic drugs. Not only that, HC-NG greatly enhanced the delivery efficiency of hydrophilic drugs, it also increased drug adhesion in the cornea through hydrophobic interactions and improved bioavailability. Thus, HC-NG can represent an efficient and effective nanocarrier for delivering a wide range of bioactive molecules into the eye after topical administration and is suitable for the treatment of both anterior and posterior segmental diseases.

Curcumin (CUR), resveratrol (RSV), ferulic acid, and the previously mentioned quercetin are natural bioactive substances with powerful antioxidant and anti-inflammatory properties that have intrigued researchers, and there is growing evidence that they have great potential to treat ocular diseases [145,146,147,148]. However, poor physicochemical and pharmacokinetic properties make effective delivery to the eye a challenge. Liu and his colleagues [93] created thermosensitive in situ nanogels by combining cationic nanostructured lipid carriers (CNLC) with a mixture of Poloxam 407 and Poloxam 188, thermosensitive gelling agents. At lower temperatures of about 5 °C, the system was a flowable liquid, though, at physiological temperatures of about 35 °C, it transformed into an unflowable gel, enhancing the retention ability and bioavailability of CUR in the aqueous humor. The nanogels were found to be nonirritating in tests and may be suitable for a variety of ocular applications. RSV has low water solubility and chemical instability. Buosi et al. [94] prepared RSV-loaded nanogels based on high-molecular-weight chitosan (HCS) crosslinked with sodium tripolyphosphate to overcome these two drawbacks. Chitosan in nanogels has been demonstrated through experiments to protect RSV from UV-induced deterioration and without cytotoxicity in human retinal pigment epithelial cells, providing an opportunity for application in the treatment of ocular diseases. Since antioxidant levels at the ocular surface have been proven to be related to corneal wound healing, Grimaudo, and her team created a micelle-nanogel that contains ferulic acid [95]. Micelles not only serve as antioxidants, they also effectively enhance the permeability of the transocular barrier, improve drug retention on the ocular surface, and promote drug absorption in the cornea [149]. Studies have revealed that micellar nanogels improve the solubility of ferulic acid and release it for up to two days in vitro, in contrast to micelles loaded with ferulic acid alone. Moreover, micellar nanogels enhanced the antioxidant property of ferulic acid, stimulated the proliferation of fibroblasts, and contributed to corneal wound healing.

## 5. Conclusions and Future Prospects

Due to the anatomical and physiological barriers of the eye, traditional ophthalmic formulations have encountered numerous challenges to topical drug delivery. Nevertheless, considerable efforts have been made to date, and new ocular drug delivery strategies have been continuously explored in the search for safer and more effective therapeutic agents. The application of nanogels is considered a promising strategy for ocular drug delivery with the dual properties of hydrogels and nanoparticles to improve ocular bioavailability and therapeutic efficacy and reduce systemic absorption and toxicity. Without effective treatment, ocular diseases such as glaucoma, cataracts, dry eye syndrome, and bacterial keratitis may ultimately result in vision loss or blindness. However, encapsulating ocular drugs in nanogels can prolong release and decrease the frequency of administration, which increases patient compliance. By responding to ocular changes in the microenvironment, including pH, temperature, and enzymes, nanogels made from various stimuli-responsive polymers can smartly control drug delivery. Multistimuli-responsive nanogels, used in IOLs and contact lenses, have demonstrated beneficial therapeutic outcomes and indicate promising clinical application prospects.

The safety of nanogels used in the eye should attract a significant amount of attention since the eye is one of the most vital and sensitive organs in the human body. The residual surfactants or unreacted monomers in nanogel preparation may be toxic. This is a problem that must be addressed in future research in order to enhance the translation of laboratory findings into practical applications. Drug toxicity and biocompatibility results for most of these articles included in this review show that there is no obvious sign of altered pharmacological effects or toxicity when nanogel formulations are used for the eye. Even though nanogels have a great deal of potential for ocular drug delivery, the vast majority of preclinical experiments have been conducted on animal models, where the proportions of the eyeball structure are quite different from those shown in people and where results are not always successfully repeated in humans. Therefore, researchers need to further evaluate the potential toxicity of nanogels during repeated and long-term application in the eye, as well as the materials used to prepare nanogels, before clinical application and commercial production. Finally, in the near future, expect more innovations in novel and dependable nanogel drugs, which will play an important role in the treatment of ocular diseases and the healthy and enjoyable lives of everyone.

## Figures and Tables

**Table 1:** Some examples of the ocular application of nanogels.

Author/Year	Applications	Polymers	Drug-Loaded	Preparations
Ilka/2018 [77]	Glaucoma	Chitosan, Sodium alginate	Timolol Maleate	Pregelation method
Cuggino/2021 [78]	Glaucoma	N-Isopropylacrylamide, Acrylic acid	Timolol Maleate	Free-radical polymerization
Abd El-Rehim/2013 [79]	Glaucoma	Polyvinylpyrrolidone, Acrylic acid	Pilocarpine	γ radiation-induced polymerization
Abdel-Rashid/2019 [80]	Glaucoma	Chitosan, Tripolyphosphate	Acetazolamide	Ionic-gelation method
Nibourg/2016 [81]	Cataract	Nanofibers, Synthetic peptides	/	Chemical crosslinking/selfassembly
Gautam/2021 [82]	Cataract	Acrylic acid, MethAcrylic acid	Sorbinil	Photo-induced aggregation
Swilem/2020 [83]	Dry eye syndrome	Polyvinylpyrrolidone, Acrylic acid	/	Ionizing-radiation method
Lin/2022 [84]	Dry eye syndrome	Lysine hydrochloride	/	Pyrolysis
Davaran/2015 [85]	Bacterial keratitis	N-Isopropylacrylamide, Methacrylic acid, Vinylpyrrolidone	Ciprofloxacin	Free-radical polymerization
Obuobi/2020 [86]	Bacterial keratitis	Deoxyribonucleic acid, L12 antimicrobial peptides	L12 antimicrobial peptides	Physical crosslinking/selfassembly
Lin/2021 [87]	Bacterial keratitis	Quercetin, Lysine	/	Pyrolysis
Kim/2014 [88]	Contact lenses	Nanodiamond-Polyethyleneimine, Chitosan	Timolol Maleate	Pyrolysis
Lee/2019 [89]	Contact lenses	N-Isopropylacrylamide, N,N′-methylenebis (acrylamide)	Timolol Maleate	Emulsion polymerization
Wang/2021 [90]	Contact lenses	Hydroxyethyl methacrylate, N-vinyl pyrrolidinone	Levofloxacin	Reflux-precipitation polymerization
Jamard/2016 [91]	Ocular Delivery	N-tert-butylacrylamide, Methylcellulose	Dexamethasone	Chemical crosslinking/selfassembly
Zoratto/2021 [92]	Ocular Delivery	Hyaluronan, Cholesterol	Tobramycin/Diclofenac sodium salt/Dexamethasone/Piroxicam	Chemical crosslinking/selfassembly
Liu/2016 [93]	Ocular Delivery	Cationic nanostructured lipid carriers, Poloxamer	Curcumin	Pregelation method
Buosi/2020 [94]	Ocular Delivery	High molecular weight chitosan, Sodium tripolyphosphate	Resveratrol	Ionic-gelation method
Grimaudo/2020 [95]	Ocular Delivery	Hyaluronan, ε-polylysine	Ferulic acid	Ionic-gelation method

## Data Availability

Not applicable.
